# Cross-Verification of COVID-19 Information Obtained From Unofficial Social Media Accounts and Associated Changes in Health Behaviors: Web-Based Questionnaire Study Among Chinese Netizens

**DOI:** 10.2196/33577

**Published:** 2022-05-31

**Authors:** Peiyi Li, Bo Chen, Genevieve Deveaux, Yunmei Luo, Wenjuan Tao, Weimin Li, Jin Wen, Yuan Zheng

**Affiliations:** 1 Department of Anesthesiology West China Hospital Sichuan University Chengdu China; 2 Laboratory of Anesthesia and Critical Care Medicine, National-Local Joint Engineering Research Centre of Translational Medicine of Anesthesiology West China Hospital Sichuan University Chengdu China; 3 The Research Units of West China (2018RU012)-Chinese Academy of Medical Sciences West China Hospital Sichuan University Chengdu China; 4 Institute of Hospital Management West China Hospital Sichuan University Chengdu China; 5 Milken Institute School of Public Health George Washington University Washington, DC United States; 6 West China Medical Publishers West China Hospital Sichuan University Chengdu China; 7 Department of Respiratory and Critical Care Medicine West China Hospital Sichuan University Chengdu China; 8 Institute of Respiratory Health, Frontiers Science Center for Disease-related Molecular Network West China Hospital Sichuan University Chengdu China; 9 President's Office West China Hospital Sichuan University Chengdu China; 10 Publicity Department West China Hospital Sichuan University Chengdu China

**Keywords:** COVID-19, pandemic, social media, behavior change, information cross-verification, eHealth literacy

## Abstract

**Background:**

As social media platforms have become significant sources of information during the pandemic, a significant volume of both factual and inaccurate information related to the prevention of COVID-19 has been disseminated through social media. Thus, disparities in COVID-19 information verification across populations have the potential to promote the dissemination of misinformation among clustered groups of people with similar characteristics.

**Objective:**

This study aimed to identify the characteristics of social media users who obtained COVID-19 information through unofficial social media accounts and were (1) most likely to change their health behaviors according to web-based information and (2) least likely to actively verify the accuracy of COVID-19 information, as these individuals may be susceptible to inaccurate prevention measures and may exacerbate transmission.

**Methods:**

An online questionnaire consisting of 17 questions was disseminated by West China Hospital via its official online platforms, between May 18, 2020, and May 31, 2020. The questionnaire collected the sociodemographic information of 14,509 adults, and included questions surveying Chinese netizens’ knowledge about COVID-19, personal social media use, health behavioral change tendencies, and cross-verification behaviors for web-based information during the pandemic. Multiple stepwise regression models were used to examine the relationships between social media use, behavior changes, and information cross-verification.

**Results:**

Respondents who were most likely to change their health behaviors after obtaining web-based COVID-19 information from celebrity sources had the following characteristics: female sex (*P*=.004), age ≥50 years (*P*=.009), higher COVID-19 knowledge and health literacy (*P=*.045 and *P*=.03*,* respectively), non–health care professional (*P*=.02), higher frequency of searching on social media (*P*<.001), better health conditions (*P*<.001), and a trust rating score of more than 3 for information released by celebrities on social media (*P=*.005). Furthermore, among participants who were most likely to change their health behaviors according to social media information released by celebrities, female sex (*P*<.001), living in a rural residence rather than first-tier city (*P*<.001), self-reported medium health status and lower health care literacy (*P*=.007 and *P*<.001*,* respectively), less frequent search for COVID-19 information on social media (*P*<.001), and greater level of trust toward celebrities’ social media accounts with a trust rating score greater than 1 (*P*≤.04) were associated with a lack of cross-verification of information.

**Conclusions:**

The findings suggest that governments, health care agencies, celebrities, and technicians should combine their efforts to decrease the risk in vulnerable groups that are inclined to change health behaviors according to web-based information but do not perform any fact-check verification of the accuracy of the unofficial information. Specifically, it is necessary to correct the false information related to COVID-19 on social media, appropriately apply celebrities’ star power, and increase Chinese netizens’ awareness of information cross-verification and eHealth literacy for evaluating the veracity of web-based information.

## Introduction

### Background

Because of the unprecedented magnitude of the COVID-19 pandemic and initial uncertainty about the virus, strategies, such as maintaining social distance and frequent hand washing, were deemed to be the most effective and feasible countermeasures [1]. In such public health crises, the general public always plays a crucial role in mitigating the spread of the disease by actively engaging in effective preventive health behaviors [2]. Thus, efficient and effective anti–COVID-19 information management in combination with public adherence to preventive health behaviors is essential for slowing the spread of the virus [3]. Social media use has increased tremendously over the last few decades because of the speed of communication, large volume of users, accessibility, and transparency [4]. Several social media platforms are available worldwide, including Facebook, Twitter, and YouTube [5], along with Chinese equivalents, such as WeChat [6], Sina Weibo [7], and TikTok [8], which offer timely updates, vivid descriptions with animated pictures, and short videos [9], and have emerged as the most preferred and actively used social media platforms among Chinese netizens [10]. Chinese netizens are defined by the China Internet Network Information Center (CNNIC) as Chinese citizens who use the internet for at least 1 hour per week, and the number has reached 1.032 billion as of December 2021 [11].

Because the pandemic put individuals at high risk of infection and created a situation of great uncertainty, individuals experienced high levels of concern and anxiety. Thus, they began to seek help through the most accessible avenues available to them, namely, social media [12], in the hope that these platforms would help them make sound decisions about their health and safety [13]. High use volume and nonphysical contact have made social media a powerful tool for facilitating the dissemination of information pertaining to COVID-19 prevention protocols and safety guidelines [14]. At the onset of the outbreak in China on January 23, 2020, there was an 87% increase in social media use [15]. These platforms also offer Chinese netizens an open and free space to make comments; interact with others; and produce, obtain, disseminate, and retransmit information about COVID-19 without extensive restrictions or censorship [16].

### Prior Work

Previous studies have found that social media can be used to disseminate health improvement measures [17], promote individual adoption of healthier behavioral patterns [18], and prevent negative health behaviors [19]. However, individuals can also be influenced to make harmful or counterproductive behavioral changes by misinformation disseminated through social media [20]. Misinformation refers to false or inaccurate information that is spread intentionally or unintentionally [21], and can be easily disseminated to large audiences on social media platforms at a very low cost [22]. The extensive COVID-19 information disseminated on social media has been extremely multifarious, with various unofficial entities engaging in producing and spreading information or misinformation ranging from hard facts to unfounded conspiracy theories [23]. In China, nearly 87% of netizens said they had encountered misinformation during the pandemic [24]. Notably, this misinformation not only causes the spread of unnecessary fears and conspiracies, but also distorts individuals’ behavioral responses to the disease [25].

Individuals with access to various sources of COVID-19 information are more likely to be knowledgeable about the correct preventive measures, which facilitates appropriate health behavioral changes [26]. Fact checking web-based information, especially that released by unofficial accounts, by finding a consensus with other official social media sources or by directly consulting physicians or specialists is a feasible approach [27]. Such cross-validation efforts help netizens perceive health issues accurately when both accurate information and misinformation coexist on social media [28]. In addition, past research has shown that people’s trust in social media accounts affects their tendency to follow preventive health information posted by that account and their decision to validate that information [29]. Therefore, it is crucial to identify vulnerable netizens who are likely to change their health behaviors based on information from unofficial social media accounts, but are also unlikely to verify that information.

### The Goals of This Study

The original contribution of this study is related to its aim to increase knowledge of the behaviors of Chinese netizens during the pandemic by addressing some gaps in the literature. In particular, this study identified the characteristics of Chinese netizens who primarily obtain COVID-19 information from unofficial social media and who are (1) more likely to change their health behaviors based on information from unofficial social media and (2) inclined to directly change their health behaviors without cross-referencing the veracity of web-based information released by unofficial sources. 

## Methods

### Setting

West China Hospital (WCH), Sichuan University, is one of the largest single-site hospitals in the world, ranking second among general hospitals in China [30]. WCH has official social media accounts on WeChat, Weibo, and TikTok that are operated by its publicity department. As of May 2020, the numbers of active followers of WCH’s social media accounts were 1,500,000 (WeChat), 495,000 (Weibo), and 421,000 (TikTok). Taking advantage of WCH’s large number of Chinese netizens based on its official social media accounts, this study distributed a web-based cross-sectional survey using convenience sampling through WCH’s official social media accounts. Data were collected through an anonymous online questionnaire from May 18 to May 31, 2020.

### Ethical Considerations

The study was approved by the Research Ethics Committee of WCH. The manuscript adhered to the reporting standards outlined by the Checklist for Reporting the Results of Internet E-Surveys (CHERRIES) and the Strengthening the Reporting of Observational Studies in Epidemiology (STROBE) guidelines [31,32].

### Study Design and Recruitment

The questionnaire was created on the online survey platform *Wen Juan Xing* (similar to Qualtrics) and generated with a URL link for dissemination. Thereafter, the URL link to access the questionnaire was posted on the social media accounts of WCH. Specifically, the followers of WCH’s social media accounts who met the inclusion criteria were invited to visit the URL link and answer the questionnaire, and were encouraged to share the link with others. Because of the nature of the questionnaire, the inclusion criteria were individuals who (1) were at least 18 years old; (2) were able to read and complete the online questionnaire independently; and (3) voluntarily agreed to participate in the survey after being provided with information about the objectives and scope of the study, as well as privacy measures and instructions for completing the survey. The privacy of each participant was protected because the questionnaire did not collect individually identifiable information. Participants were free to exit from the questionnaire at any point. All participants were only allowed to submit 1 survey response, which was verified through *Wen Juan Xing* by automatically verifying that each participant’s IP address only submitted 1 response.

### Instruments and Measures

#### Instruments

The authors initially developed a questionnaire that contained 21 questions based on a literature review of relevant studies, as well as World Health Organization materials on COVID-19 [33,34] and the COVID-19 Protection Manual [35], and it was presented in Mandarin Chinese. To ensure its readability, first, the researchers stopped pedestrians at a central intersection and asked if they would be willing to answer the survey. Then, the questionnaire was modified according to the respondents’ feedback regarding any ambiguities or areas of confusion.

To ensure the validity of the questionnaire, 20 experts from different fields were selected from the Sichuan Provincial health service system, including respiratory physicians, epidemiologists, medical informaticists, and health care policy-makers. The questionnaire was evaluated by the panel of experts to validate its content with intended constructs and theories. The content validity of the questionnaire was assessed by the item-level content validity index (CVI), which was measured on a 4-point Likert scale, including different parameters such as relevance, clarity, simplicity, and ambiguity [36]. Items with a CVI >0.8 were retained, and context-specific adjustments were made according to the feedback provided by the experts. As a result, the final questionnaire consisted of 17 questions, and of these, 10 were single choice, 2 were multiple choice, and 5 were ranking questions (Multimedia Appendix 1). After the questionnaire was revised according to the experts, 30 Chinese netizens were randomly selected to read the survey. All feedback was used to adjust the survey, including rectification and clarification of words or phrases. Finally, it included 4 subsets of questions described in the following sections.

#### Sociodemographic Information

A set of sociodemographic variables was collected in the first section of the questionnaire, including gender, age (referenced from the categorization by the National Bureau of Statistics of China), educational status, occupation (referenced from the standard occupational classification in China [37]), living area (classified by the National Statistics Bureau), perceived health status, and self-assessed health literacy.

#### Social Media Use and Trust Rating

Social media use was measured by the amount of time (in hours) spent on social media per day and the frequency of searching for information related to COVID-19. A multiple-choice question was asked about which of the 5 types of accounts were preferred when searching for information about COVID-19 on social media. To measure the trustworthiness of a specific source of web-based information on social media, the participants were asked to rate the perceived trustworthiness of each type of information source using a 5-point Likert scale from 1 (least trustworthy) to 5 (most trustworthy).

#### Basic Knowledge of COVID-19

Participants’ basic knowledge of COVID-19 was evaluated using 4 questions developed based on the COVID-19 Protection Manual (China Mainland Version, January 2020), including 1 multiple-choice question related to COVID-19 transmission and 3 single-choice questions centered around the proper use of masks. Each correct answer was assigned 1 point, and incorrect answers were assigned 0 points for a maximum of 6 points.

#### Behavioral Changes and Cross-Verification of Information

To measure whether the individuals would change their health behaviors, participants were asked, “Did you change health behaviors based on the COVID-19 information on social media?” with answer options “Yes” and “No.” Subsequently, a question (“Did you cross-verify the authenticity of COVID-19 information on social media?”) was asked to identify the participants’ cross-verification behavior, with answer options “Yes” and “No.” Although the Likert approach is more accurate in capturing the variation and degree of behavioral change and cross-validation, the criterion here was the presence or absence of respondents’ actual action; thus, binary measurement was used for analysis.

### Statistical Analysis

Descriptive statistics were used to assess all sociodemographic characteristics of the participants. Frequency and case-weighted percentages were calculated to describe sociodemographic parameters and level distributions among participants. Differences in characteristics between groups were investigated with descriptive analyses performed according to the characteristics of the data, including the chi-square test and Kruskal-Wallis test.

Multiple stepwise regression was used to examine the association between the independent and dependent variables [38]. Specifically, the authors first included sociodemographic information, social media use, sources of information on social media, and the trust rating as control variables for Model 1, with the dependent variable “COVID-19 knowledge score.” Then, participants who obtained web-based information from less reliable sources, namely, celebrity social media accounts, were further evaluated in Models 2 and 3. In Model 2, the COVID-19 knowledge score was introduced with the dependent variable “behavior change.” Finally, those participants who changed or did not change their behaviors were introduced as a control variable in Model 3, with the dependent variable “information cross-verification.” Key outcomes were presented according to standardized regression coefficients, adjusted odds ratios (aORs), and 95% CIs, and were analyzed using SPSS version 23 (IBM Corp). A *P* value <.05 was considered to be statistically significant.

## Results

### Sample Characteristics

A total of 15,055 Chinese netizens completed the survey, and 14,509 responses were included in the study after incomplete survey responses were excluded (14,509/15,055, 96.4%). The descriptive analysis shown in Table 1 indicates that socioeconomic attributes varied by age group. Among the respondents, 20.7% (3008/14,509) were male and 42.4% (6151/14,509) were between 30 and 39 years old. Furthermore, more than half (9792/14,509, 67.5%) of the participants had a bachelor’s degree or higher, while 5.9% (849/14,509) reported that they lived in rural areas. Moreover, older participants were more likely to report a poor health status and low health care literacy. Furthermore, younger participants were generally more active in web-based activities (*P*<.001). In contrast, older respondents (≥40 years) used social media more often to seek COVID-19 information than other age groups (*P*<.001).

**Table 1:** Demographic characteristics of the participants.

Characteristic			Total (N=14,509), n (%)		Age groups (years)									*P* value
					18-29 (N=5723), n (%)		30-39 (N=6151), n (%)		40-49 (N=1714), n (%)		≥50 (N=921), n (%)			
**Gender**													<.001^a^	
	Male	3008 (20.7)		1297 (22.7)		1139 (18.5)		349 (20.4)		223 (24.2)				
	Female	11,501 (79.3)		4426 (77.3)		5012 (81.5)		1365 (79.6)		698 (75.8)				
**Educational status**													<.001^b^	
	Junior high school or below	407 (2.8)		100 (1.7)		89 (1.4)		118 (6.9)		100 (10.9)				
	High school	1242 (8.6)		368 (6.4)		420 (6.8)		240 (14.0)		214 (23.2)				
	Junior college	3068 (21.1)		1218 (21.3)		1115 (18.1)		439 (25.6)		296 (32.1)				
	Undergraduate degree	7685 (53.0)		3182 (55.6)		3480 (56.6)		742 (43.3)		281 (30.5)				
	Master’s degree or above	2107 (14.5)		855 (14.9)		1047 (17.0)		175 (10.2)		30 (3.3)				
**Occupation**													<.001^a^	
	Student	1661 (11.4)		1637 (28.6)		22 (0.4)		1 (0.1)		1 (0.1)				
	Staff member in the government	2436 (16.8)		656 (11.5)		1282 (20.8)		367 (21.4)		131 (14.2)				
	Health care provider	2192 (15.1)		1075 (18.8)		879 (14.3)		183 (10.7)		55 (6.0)				
	Staff member in a company	3258 (22.5)		978 (17.1)		1737 (28.2)		463 (27.0)		80 (8.7)				
	Self-employed entrepreneur	965 (6.7)		270 (4.7)		518 (8.4)		142 (8.3)		35 (3.8)				
	Other	3997 (27.5)		1107 (19.3)		1713 (27.8)		558 (32.6)		619 (67.2)				
**Current residence**													<.001^a^	
	First-tier city	549 (3.8)		280 (4.9)		202 (3.3)		47 (2.7)		20 (2.2)				
	Second-tier city	9133 (62.9)		3562 (62.2)		4078 (66.3)		980 (57.2)		513 (55.7)				
	Other city	3978 (27.4)		1444 (25.2)		1646 (26.8)		564 (32.9)		324 (35.2)				
	Rural area	849 (5.9)		437 (7.6)		225 (3.7)		123 (7.2)		64 (6.9)				
**Perceived health status**													<.001^b^	
	Good	9251 (63.8)		4106 (71.7)		3679 (59.8)		962 (56.1)		504 (54.7)				
	Medium	4515 (31.1)		1393 (24.3)		2153 (35.0)		643 (37.5)		326 (35.4)				
	Poor	743 (5.1)		224 (3.9)		319 (5.2)		109 (6.4)		91 (9.9)				
**Health care literacy**													<.001^b^	
	High	5978 (41.2)		2589 (45.2)		2373 (38.6)		666 (38.9)		350 (38.0)				
	Medium	7090 (48.9)		2598 (45.4)		3155 (51.3)		871 (50.8)		466 (50.6)				
	Low	1441 (9.9)		536 (9.4)		623 (10.1)		177 (10.3)		105 (11.4)				
**Time spent on social media per day (hours)**													<.001^b^	
	≤1	797 (5.5)		266 (4.6)		327 (5.3)		119 (6.9)		85 (9.2)				
	>1 to ≤3	7108 (49.0)		2435 (42.5)		3233 (52.6)		925 (54.0)		515 (55.9)				
	>3 to ≤5	4376 (30.2)		1916 (33.5)		1737 (28.2)		485 (28.3)		238 (25.8)				
	>5 to ≤7	1418 (9.8)		670 (11.7)		565 (9.2)		122 (7.1)		61 (6.6)				
	>7	810 (5.6)		436 (7.6)		289 (4.7)		63 (3.7)		22 (2.4)				
**Frequency of browsing information related to COVID-19**													<.001^b^	
	Rarely	573 (3.9)		267 (4.7)		230 (3.7)		47 (2.7)		29 (3.1)				
	Sometimes	2107 (14.5)		922 (16.1)		912 (14.8)		177 (10.3)		96 (10.4)				
	Often	11,829 (81.5)		4534 (79.2)		5009 (81.4)		1490 (86.9)		796 (86.4)				

^a^Chi-square test.

^b^Kruskal-Wallis test.

### Use of and Trust in Various Social Media Sources and COVID-19 Knowledge

Table 2 presents Chinese netizens’ use of and trust in different sources of web-based information on social media. The participants sought COVID-19 information through a variety of social media channels, favoring professional news media (12,706/14,509, 87.6%), government agencies (12,255/14,509, 84.5%), and health care media (8124/14,509, 56.0%), followed by hospital institutions (7107/14,509, 49.0%) and celebrities (4017/14,509, 27.7%). The trust scores for different sources were averaged to generate an overall score, which indicated that the most trusted source of COVID-19 information was hospital institutions (mean 4.52, SD 0.69), followed by government agencies (mean 4.46, SD 0.76), professional news media (mean 4.18, SD 0.79), health care media (mean 3.86, SD 0.87), and celebrities (mean 3.21, SD 1.07).

Table 3 shows that the sample of Chinese netizens had a high level of knowledge of preventive measures against COVID-19, but few participants lacked awareness of COVID-19 “airborne” transmission (correct option: 8990/14,509, 62.0%). In addition, 5.0% (723/14,509) chose the incorrect types of masks for preventing COVID-19 and 7.2% (1052/14,509) selected incorrect options for mask use methods. In total, 3.1% (448/14,509) of the respondents perceived “drinking alcohol,” “sauna or steaming,” and “rinsing with light saltwater’’ as feasible COVID-19 countermeasures.

**Table 2:** Sources of COVID-19 information on social media and source trust scores.

Variable				Total (N=14,509)		Age groups (years)									*P* value
						18-29 (N=5723)		30-39 (N=6151)		40-49 (N=1714)		≥50 (N=921)			
**Social media outlets used to search for COVID-19 information, n (%)**															
	**Government agencies**												.04^a^		
		Yes	12,255 (84.5)		4773 (83.4)		5241 (85.2)		1460 (85.2)		781 (84.8)				
		No	2254 (15.5)		950 (16.6)		910 (14.8)		254 (14.8)		140 (15.2)				
	**Professional news media**												<.001^a^		
		Yes	12,706 (87.6)		5024 (87.8)		5432 (88.3)		1483 (86.5)		767 (83.3)				
		No	1803 (12.4)		699 (12.2)		719 (11.7)		231 (13.5)		154 (16.7)				
	**Health care media**												<.001^a^		
		Yes	8124 (56.0)		3570 (62.4)		3357 (54.6)		812 (47.4)		385 (41.8)				
		No	6385 (44.0)		2153 (37.6)		2794 (45.4)		902 (52.6)		536 (58.2)				
	**Hospital institutions**												<.001^a^		
		Yes	7107 (49.0)		2743 (47.9)		2964 (48.2)		911 (53.2)		489 (53.1)				
		No	7402 (51.0)		2980 (52.1)		3187 (51.8)		803 (46.8)		432 (46.9)				
	**Celebrities**												<.001^a^		
		Yes	4017 (27.7)		1671 (29.2)		1595 (25.9)		447 (26.1)		304 (33.0)				
		No	10,492 (72.3)		4052 (70.8)		4556 (74.1)		1267 (73.9)		617 (67.0)				
**Trust score for different sources of information, mean (SD)**															
	Government agencies^b^		4.46 (0.76)		4.49 (0.75)		4.46 (0.76)		4.39 (0.77)		4.40 (0.82)		<.001^c^		
	Professional news media^d^		4.18 (0.79)		4.18 (0.80)		4.21 (0.77)		4.15 (0.79)		4.11 (0.87)		.002^c^		
	Health care media^e^		3.86 (0.87)		3.87 (0.88)		3.89 (0.85)		3.80 (0.86)		3.78 (0.89)		<.001^c^		
	Hospital institutions^f^		4.52 (0.69)		4.53 (0.67)		4.53 (0.68)		4.50 (0.71)		4.51 (0.74)		.76^c^		
	Celebrities^g^		3.21 (1.07)		3.27 (1.07)		3.18 (1.04)		3.15 (1.10)		3.14 (1.13)		<.001^c^		

^a^Chi-square test.

^b^Government agencies, such as the Chinese State Council, which often serve as the voice of official or administrative institutions.

^c^Kruskal-Wallis test.

^d^Professional news media outlets, such as Sina Release, which focus on instant news reporting in the professional domain.

^e^Health care institutions, such as the US Centers for Disease Control and Prevention, which often cover trends in the medical field and issue public health advisories.

^f^Hospital institutions, such as West China Hospital accounts, which disseminate prevention and treatment information.

^g^Celebrities who have a large number of social media followers and overall social and consumer influence [39].

**Table 3:** Participants’ knowledge about COVID-19.

Questions and responses		Value (N=14,509), n (%)
**Modes of transmission^a^**		
	Droplet (correct option)	14,214 (98.0)
	Airborne (correct option)	8990 (62.0)
	Close contact (correct option)	12,353 (85.1)
**Which of the following is not suitable for preventing COVID-19 in the choice of masks?**		
	Cloth mask (correct option)	13,786 (95.0)
	Disposable medical mask	254 (1.8)
	Medical-surgical mask	292 (2.0)
	N95 protective mask	177 (1.2)
**Which of the following statements is incorrect about the use of masks?**		
	If conditions permit, populations in dense areas should change their disposable masks around 4 hours	522 (3.6)
	Once contaminated, it should be replaced as soon as possible	291 (2.0)
	Avoid touching the inner face of the mask with your hands	239 (1.6)
	Cotton masks resist the coronavirus better than medical masks (correct option)	13,457 (92.8)
**Which of the following measures is recommended by the Chinese Centers for Disease Control to protect against COVID-19 transmission?**		
	Rinsing with light saltwater	148 (1.0)
	Sauna or steaming	102 (0.7)
	Drinking alcohol	198 (1.4)
	Wearing masks (correct option)	14,061 (96.9)

^a^There were multiple correct options.

### Multivariable Analyses of the COVID-19 Knowledge Score, Behavioral Change, and Cross-Verification

We identified the following groups as having a higher likelihood of obtaining accurate COVID-19 preventive information: female participants (*P*<.001), those aged 30-39 or 40-49 years (both *P*<.001), health care workers (*P*=.01), those living in cities (*P*<.001), those having a poor health status (*P*<.001), those having an online time of 1-5 hours (*P*=.005) or more than 7 hours per day (*P*=.02), and those having a high frequency of searching for COVID-19 information on social media (*P*=.001) (Table 4). In addition, those with high trust in web-based information from professional media and hospital institutions with a trust rating score more than 3 had a higher level of COVID-19 prevention knowledge (*P*<.05). On the other hand, those with trust rating scores of 5 for online information released from celebrities’ social media accounts were more likely to have insufficient COVID-19 preventive knowledge (*P*<.001).

Among 4017 participants who searched for COVID-19 information on celebrities’ social media accounts, those who were female, were aged ≥50 years, were non–health care workers, had a higher perceived health condition and health literacy, and had a higher frequency of searching had greater odds of behavioral changes based on COVID-19 web-based information (Table 5). Additionally, having high COVID-19 knowledge was associated with significantly higher odds of behavioral changes (aOR 1.085, 95% CI 1.036-1.191; *P*=.045). Those with high trust rating scores of more than 3 for social media information from celebrities were more likely to change their behaviors according to online information (*P*<.001).

In terms of subgroups who searched for COVID-19 web-based information released by celebrities and who were more likely to change their health behaviors, we found that being female (aOR 0.767, 95% CI 0.544-0.928; *P*<.001), having a self-reported medium health condition (aOR 0.789, 95% CI 0.664-0.939; *P*=.007), having a self-reported medium and low health literacy (aOR 0.596, 95% CI 0.505-0.703; *P*<.001 and aOR 0.441, 95% CI 0.323-0.600; *P*<.001, respectively), and having a high trust score of more than 1 for online information released by celebrities (*P*<.05) were associated with lower odds of information cross-validation (Table 5). Nevertheless, participants who resided in first-tier cities (aOR 1.455, 95% CI 1.260-2.144; *P*<.001) and those who often browsed internet information related to COVID-19 (aOR 3.239, 95% CI 1.632-6.788; *P*<.001) had greater odds of performing COVID-19 information cross-validation.

**Table 4:** Multiple linear regression results of the association of COVID-19 knowledge with demographic characteristics and social media use.

Variable					COVID-19 knowledge score						
					Coefficient			Standard error			*P* value
Gender (female vs male)					0.172			0.018			<.001
**Age (years, vs 18-29 years)**											
	30-39			0.075			0.017			<.001	
	40-49			0.108			0.025			<.001	
	≥50			−0.138			0.032			<.001	
**Educational status (vs junior high school or below)**											
	High school			0.050			0.049			.30	
	Junior college			0.052			0.046			.26	
	Undergraduate degree			0048			0.046			.30	
	Master’s degree or above			−0.016			0.049			.75	
**Occupation (vs student)**											
	Staff member in the government			−0.003			0.030			.91	
	Health care provider			0.073			0.029			.01	
	Staff member in a company			0.006			0.029			.84	
	Self-employed entrepreneur			0.002			0.037			.95	
	Other			0.053			0.028			.06	
**Current residence (vs rural area)**											
	First-tier city			0.158			0.037			<.001	
	Second-tier city			0.160			0.039			<.001	
	Other city			0.168			0.047			<.001	
**Perceived health status (vs good)**											
	Medium			0.001			0.016			.96	
	Poor			0.131			0.033			<.001	
**Medical information literacy (vs high)**											
	Medium			0.019			0.016			.22	
	Low			−0.026			0.027			.33	
**Time spent on social media (hours, vs ≤1 hour)**											
	>1 to ≤3			0.111			0.032			.001	
	>3 to ≤5			0.093			0.033			.005	
	>5 to ≤7			0.060			0.038			.11	
	>7			0.101			0.043			.02	
**Frequency of browsing information related to COVID-19 (vs rarely)**											
	Sometimes			0.305			0.040			<.001	
	Often			0.379			0.037			<.001	
**Sources of information about COVID-19 on social media**											
	Government agencies (no vs yes)			0.188			0.020			<.001	
	Professional news media (no vs yes)			0.245			0.022			<.001	
	Health care media (no vs yes)			0.063			0.015			<.001	
	Hospital institutions (no vs yes)			0.094			0.015			<.001	
	Celebrities (no vs yes)			0.087			0.017			<.001	
**Trust rating score for different sources of information**											
	**Government agencies (vs 1)**										
		2	—^a^			—			N/A^b^		
		3	—			—			N/A		
		4	—			—			N/A		
		5	—			—			N/A		
	**Professional news media (vs 1)**										
		2	0.064			0.123			.60		
		3	0.384			0.116			.001		
		4	0.364			0.115			.002		
		5	0.421			0.116			<.001		
	**Health care media (vs 1)**										
		2	−0.037			0.085			.67		
		3	−0.127			0.080			.11		
		4	−0.135			0.080			.09		
		5	−0.183			0.081			.06		
	**Hospital institutions (vs 1)**										
		2	−0.163			0.143			.25		
		3	0.256			0.122			.04		
		4	0.376			0.119			.002		
		5	0.444			0.119			<.001		
	**Celebrities (vs 1)**										
		2	0.000			0.032			.99		
		3	0.025			0.029			.39		
		4	−0.045			0.030			.14		
		5	−0.120			0.035			.001		

^a^The corresponding variable has not been included in the final multiple regression model.

^b^N/A: not applicable.

As shown in Table 5, among those who were less likely to change their behaviors according to web-based information released by celebrities, those who were female (aOR 1.419, 95% CI 1.050-1.921; *P*=.02) and who more frequently browsed internet information related to COVID-19 (aOR 4.077, 95% CI 1.906-9.742; *P*<.001) had higher odds of cross-validating COVID-19 information. However, participants who self-reported medium and low health literacy (aOR 0.614, 95% CI 0.476-0.791; *P*<.001 and aOR 0.529, 95% CI 0.338-0.822; *P*=.005, respectively) were less likely to check the veracity of COVID-19 information when determining their personal behaviors.

**Table 5:** Multiple logistic regression results of the association between behavior change and verification.

Variable			Behavior change					Information verification (among netizens searching web-based COVID-19 information released by celebrities)									
			Change vs no change					Behavior change group					No behavior change group				
			aOR^a^ (95% CI)		*P* value		Verify vs not verify, aOR (95% CI)			*P* value		Verify vs not verify, aOR (95% CI)			*P* value		
COVID-19 knowledge score			1.085 (1.036-1.191)		.045		—^b^			N/A^c^		—			N/A		
Gender (female vs male)			1.301 (1.085-1.556)		.004		0.767 (0.544-0.928)			<.001		1.419 (1.050-1.921)			.02		
**Age (years, vs 18-29 years)**																	
	30-39	1.161 (0.981-1.374)		.08		—			N/A		—			N/A			
	40-49	1.284 (0.998-1.660)		.054		—			N/A		—			N/A			
	≥50	1.519 (1.116-2.089)		.009		—			N/A		—			N/A			
**Educational status (vs junior high school or below)**																	
	High school	—		N/A		0.695 (0.386-1.233)			.22		—			N/A			
	Junior college	—		N/A		0.786 (0.452-1.345)			.39		—			N/A			
	Undergraduate degree	—		N/A		0.613 (0.357-1.034)			.07		—			N/A			
	Master’s degree or above	—		N/A		0.725 (0.409-1.268)			.27		—			N/A			
**Occupation (vs student)**																	
	Staff member in the government	1.053 (0.779-1.425)		.74		—			N/A		—			N/A			
	Health care provider	0.721 (0.550-0.943)		.02		—			N/A		—			N/A			
	Staff member in a company	1.130 (0.850-1.499)		.40		—			N/A		—			N/A			
	Self-employed entrepreneur	1.140 (0.797-1.639)		.48		—			N/A		—			N/A			
	Other	1.045 (0.792-1.378)		.75		—			N/A		—			N/A			
**Current residence (vs rural area)**																	
	First-tier city	—		N/A		1.455 (1.260-2.144)			<.001		—			N/A			
	Second-tier city	—		N/A		1.281 (0.899-1.419)			.06		—			N/A			
	Other city	—		N/A		0.799 (0.526-1.200)			.28		—			N/A			
**Perceived health status (vs good)**																	
	Medium	1.046 (0.893-1.226)		.58		0.789 (0.664-0.939)			.007		—			N/A			
	Poor	0.578 (0.419-0.801)		<.001		0.770 (0.509-1.167)			.22		—			N/A			
**Medical information literacy (vs high)**																	
	Medium	0.718 (0.454-0.956)		<.001		0.596 (0.505-0.703)			<.001		0.614 (0.476-0.791)			<.001			
	Low	0.845 (0.570-0.989)		.03		0.441 (0.323-0.600)			<.001		0.529 (0.338-0.822)			.005			
**Time spent on social media (hours, vs ≤1 hour)**																	
	>1 to ≤3	—		N/A		1.156 (0.741-1.790)			.52		—			N/A			
	>3 to ≤5	—		N/A		0.809 (0.514-1.262)			.35		—			N/A			
	>5 to ≤7	—		N/A		1.258 (0.770-2.044)			.36		—			N/A			
	>7	—		N/A		1.009 (0.602-1.683)			.97		—			N/A			
**Frequency of browsing information related to COVID-19 (vs rarely)**																	
	Sometimes	1.379 (0.827-2.295)		<.001		1.077 (0.458-1.786)			.92		1.545 (0.675-3.885)			.33			
	Often	2.477 (1.541-3.974)		<.001		3.239 (1.632-6.788)			<.001		4.077 (1.906-9.742)			.001			
**Trust rating score for different sources of information (celebrities, vs 1)**																	
	2	1.043 (0.668-1.617)		.85		0.803 (0.681-0.939)			.04		—			N/A			
	3	1.330 (0.889-1.972)		.16		0.518 (0.374-0.777)			<.001		—			N/A			
	4	1.771 (1.182-2.629)		.005		0.625 (0.322-0.909)			<.001		—			N/A			
	5	2.497 (1.630-3.794)		<.001		0.386 (0.107-0.519)			<.001		—			N/A			

^a^aOR: adjusted odds ratio.

^b^The corresponding variable has not been included in the final multiple regression model.

^c^N/A: not applicable.

## Discussion

### Principal Findings

#### Social Media Use and COVID-19 Knowledge

Overall, with the advancement of smart device technology, the use of the internet has penetrated various age groups. More than 90% of those investigated reported surfing social media for more than 1 hour per day, including middle-aged and older participants. The findings also showed that social media use and the credibility of web-based information among different age groups varied. The age gap should be considered as much as possible in broadening the diffusion of preventive measures for COVID-19 via social media platforms. The results also indicated that “frequency” had a more significant impact on COVID-19 literacy than the length of time spent using social media. In other words, “how often” individuals consulted social media directly, rather than “how long,” had a strong relationship with preventive behaviors [12]. Since frequency may be a direct indicator of motivation for various types of social media use, such as self-expression, social learning, social comparison, and filtering [40], this study cautiously suggests that the frequency of social media use may be a more essential predictor of social media effects than time spent using social media [41].

Similar to a prior study that found that women had higher COVID-19 literacy [12], this survey detected that female netizens self-reported engaging in more correct preventive behaviors than male netizens. This finding may be explained by women usually having higher levels of disease knowledge and health care literacy than men [42]. A previous study also suggested that women were more sensitive to and interested in health information on social media [43], which may be another reason for their higher COVID-19 literacy, since females seem to search the internet more frequently for COVID-19 information [44]. Alternatively, the gender difference may be partly attributed to the self-reported health literacy bias in this study and previous studies. An objective measurement tool for health literacy, rather than self-ratings, is warranted to examine the gender disparity in future research. However, in our study, current residence was a direct indicator of higher COVID-19 literacy, whereas education level was nonsignificant. This inconsistency could be explained by the fact that those in rural areas usually have lower education levels [45]. These results highlight the need to pay attention to populations in remote regions in order to prevent the deterioration of their health outcomes from causing the education level to fall behind again, thus resulting in a vicious cycle [46,47].

Differing levels of trust in 5 web-based information sources on social media were found to be another significant predictor of preventive behaviors. For web-based information released by professional media and hospital institutions, higher trust was associated with a positive relationship with COVID-19 literacy. However, Chinese netizens with high trust in web-based information released by celebrities seemed to have less COVID-19 knowledge. The results indicate that the accuracy of COVID-19 information from individual and unofficial social media accounts, including those of movie stars and singers, deserves more attention than official social media accounts in terms of the effect on preventive measures, particularly for Chinese netizens. Celebrities were more influential in disseminating the related information via social media platforms, especially among young Chinese netizens [48]. The higher incidence of insufficient COVID-19 knowledge among followers of celebrities reflects the need to cross-reference web-based information released by unofficial sources with information from official sources.

#### Behavioral Changes and Social Media Use

The results showed that women were more likely than men to change their health care behaviors according to web-based information released by unofficial accounts. Women may be more sentimental and sensitive, may experience more severe stress and anxiety during the pandemic [49], and may use the internet and social media more frequently to search for related information [50]. Therefore, the authors cautiously conclude that concerns regarding this pandemic may accelerate women’s health-related behavioral changes [51]. Additionally, non–health care workers were more inclined to change their behaviors after obtaining web-based COVID-19 information. Health care workers may choose to consult academic articles before making decisions about health care behaviors. Moreover, the higher possibility of behavioral changes based on social media information among those older than 50 years may be due to the presence of more health concerns and stronger emphasis on health among these age groups [52].

Additionally, social media use frequency had a significant relationship with Chinese netizens’ adoption of web-based health care advice and changes to their preventive behaviors. Thus, “frequency” may be a more significant predictor of social media effects. Social media use frequency should therefore be an effective strategy for public health promotion, especially when countries are confronted with COVID-19 vaccination hesitancy [53]. Furthermore, the authors also detected that the possibility of behavioral change was higher if netizens had higher trust in information from celebrities’ social media outlets. Celebrities and public figures have long been shown to be important influencers of human behavior due to various proposed psychological, social, and biological mechanisms [54].

The relationship between health literacy and health behaviors has been widely recognized [55,56]. Consistent with a previous study, in this survey, the self-reported higher health literacy group seemed to change their behaviors, while less health literacy netizens were less likely to change their preventive behaviors based on information from social media as a result of a deficiency in basic medical knowledge associated with their education level. Similarly, the odds of behavioral change increased as individuals’ COVID-19 knowledge increased, which may underscore that populations that have poor preventive knowledge are more likely to be stubborn and insist on their own perceptions of effective approaches to combat COVID-19. Thus, the knowledge gap should be considered to the greatest extent possible when using social media to publicize pandemic countermeasures [57]. Of note, the aforementioned disparities in behavioral change should be carefully considered since those netizens took web-based information from celebrities as an effective avenue for the dissemination of information during the pandemic.

#### Cross-Verification of Social Media Information

Although social media–based information may help specific groups improve their ability to deal with the pandemic, individuals may also take risks in their use of web-based resources, because web-based information released by individual accounts is not always accurate [58,59]. The survey findings can help identify vulnerable netizens who are likely to change their health behaviors according to less accurate web-based information without cross-verifying its accuracy and can provide insightful implications to promote better use of social media in the fight against COVID-19 [60].

As previous research has illustrated, health literacy has been underestimated, and more emphasis should be placed on it during the pandemic [61,62]. The positive effect of health literacy on the cross-verification of web-based information suggests that health literacy plays a fundamental role during the pandemic. Remarkably, the results also revealed that among the behavioral change groups, women were less likely than men to verify the veracity of web-based information. This finding is notable since the survey also found that female netizens had higher COVID-19 preventive knowledge and engaged in more preventive behaviors than men. Thus, the authors argue that compared with health literacy, eHealth literacy or digital literacy, which is defined as “the capacity for individuals to seek, find, understand, and appraise health information from electronic sources,” might have a greater effect on the awareness of web-based information verification [63,64]. Even netizens who have high health literacy and COVID-19 knowledge may lack the eHealth literacy to be aware of, verify, and evaluate the veracity of COVID-19 web-based information posted by celebrities. Men have been reported to have higher digital literacy than women in relation to internet-based information [64]. Similarly, the low cross-verification among netizens living in rural areas extends the previous statement that there are distinct socioeconomic disparities in eHealth literacy [65]. Solving issues around limited digital health literacy, however, would benefit gender and residence disparities across cross-verification preferences in efforts to contain COVID-19 [66].

Furthermore, the high-frequency use of social media and search for information, rather than the time spent on social media, fostered the ability of groups to cross-verify information. This phenomenon was more commonly associated with the current practices of social media companies using algorithms that repeatedly drive similar content to users based on what they have recently browsed [67]. These algorithms reinforce COVID-19 misinformation for netizens who are incessantly immersed in social media and isolate them from reports on legitimate scientific evidence. Specifically, Chinese netizens who engage less frequently with social media should be encouraged to verify COVID-19 information through multiple searches for official internet sources.

The importance of cross-referencing was heavily based upon the likely veracity of the information obtained. In this context, web-based information released from official social media accounts, such as the government, the Centers for Disease Control and Prevention (CDC), and hospital institutions, is likely highly accurate, while that from individual social media accounts may be inaccurate and thus even more in need of cross-verification with official sources. Notably, in our research, Chinese netizens who trusted web-based COVID-19 information released by celebrities usually conducted little cross-verification of web-based information before changing their behaviors. Even worse, this survey found that netizens who highly trusted web-based information posted by celebrities were less knowledgeable of COVID-19 preventive measures and more likely to change their health care behaviors based on that online information. According to a Twitter survey, during the pandemic, the tweets of celebrities and politicians related to COVID-19 outperformed those of health and scientific institutions [68]. Many of the followers of celebrities, movie stars, and singers, also called blind adherents, trust everything said by their “idols.” Celebrities should be aware of their social impact and foster positive values, including delivering more credible news and dispelling rumors, which may be helpful in controlling the pandemic [69,70]. Additionally, the government should act to raise fans’ awareness of misinformation on social media and increase their eHealth literacy to improve their ability to verify web-based information related to COVID-19.

### Policy Implications

The pandemic is accompanied by an infodemic that involves the abundant and uncontrolled spread of potentially harmful misinformation, mainly produced by unofficial social media accounts [71]. As cross-referencing of internet information via different released channels is perceived to be effective for identifying accurate information, netizens who lack awareness of such information verification are a vulnerable population among those worst affected by the COVID-19 infodemic [72]. This study identified the characteristics of that vulnerable population and proposes the following measures to target them. Digital health or eHealth literacy can improve netizens’ capacity to search, compare, and take the best advantage of web-based information [73]. The government should establish programs to improve netizens’ eHealth literacy and strengthen their capacities to obtain, read, understand, and assess health care information so that they can use web-based COVID-19 information appropriately [74]. Moreover, natural language processing models and artificial intelligence (AI)-based approaches, including AI-augmented lifelong learning and AI-assisted translation, simplification, summarization, and filtering, may have the general advantages of building and enhancing netizens’ levels of digital or eHealth literacy [75,76]. Referencing Eysenbach’s fourth pillar of infodemic management, this study also suggests that data and information flow patterns on social media should be continuously monitored and analyzed [77]. Outbreaks of misinformation, rumors, and falsehoods can thus be detected immediately and countered with facts or other interventions, such as flagging or removing the content from social media platforms and decreasing the dissemination of negative information and panic among Chinese netizens [20,72]. Finally, the private accounts of celebrities should receive more relative attention since they have powerful appeal among netizens. Celebrities could help by providing valuable health information and convincing their fans to follow appropriate preventive COVID-19 measures and be vaccinated.

### Strengths, Limitations, and Future Research

This study has several strengths. First, the sample was relatively large and widely representative, which provided the opportunity for accurate examination of potential variations. Moreover, this study extends the current literature on the characteristics of Chinese netizens who are likely to change their health behaviors according to unofficial web-based information, but seldom conduct cross-verification. As countries across the world continue to battle the pandemic and confront increased use of social media for health information dissemination, similar research in the infodemic management field is expected.

Despite its strengths, several limitations of our study should be acknowledged. First, it included only 3 WCH social media platforms and people who had access to the internet and electronic devices, thereby excluding people who did not. Additionally, since this was a cross-sectional study conducted between May 18 and May 31, 2020, the results may not be generalizable and thus may fail to capture changes over time due to rapid social development. The online survey had very low response rates among older people. Considering the low use of the internet among older groups, further studies should focus on the use of traditional media for older people during the pandemic. Moreover, the self-designed questionnaire failed to evaluate the actual age, obtain a more detailed educational degree, and use a 1-5 scale of medical knowledge, which would have allowed for the collection of more specific information from the respondents. In addition, the internal validity may be an issue because WCH social media followers were encouraged to distribute the questionnaire to their relatives and friends who met the inclusion criteria. However, considering that the questionnaire items did not involve any individual interests and emphasized voluntary and uncompelled survey participation, unintentional bias associated with participant relationships was a remote possibility. Moreover, the study was completely voluntary, so the characteristics of individuals who would actively choose to participate should be considered since self-reported health status and literacy levels are highly subjective. Similarly, the study could not accurately predict netizens’ health behaviors based on self-reported behavioral change and cross-verification. However, it provides a preliminary analysis and clarifies associations between various characteristics.

Additionally, the behavioral change tendencies included in this study are not necessarily positive or negative because the survey could not discern what information a change was in response to and whether it was an effective change. Moreover, information verification is difficult to measure and is detrimental only when the information is inaccurate. Therefore, further studies regarding verification strategies are necessary. Furthermore, the sample included many more individuals with high education levels and netizens from urban areas. Future studies should include netizens with less education and those who live in rural areas to facilitate the generalizability of the findings. Moreover, this cross-sectional study focused mainly on investigating phenomena, and the barriers, facilitators, and causal loops for behavioral change and cross-verification were not included. Further research is necessary to explore what motivates individuals’ social media use, as well as barriers to and facilitators of the validation of web-based information. Finally, with the increasing popularity of social media, people’s health literacy and eHealth literacy have been continuously improving over the last few years, and future research with a wider time span could be conducted to investigate changes in cross-verification behaviors.

### Conclusions

In general, this study made the first attempt to examine whether cross-verification was implemented before Chinese netizens engaged in changes related to health behavior–based information on unofficial social media. The study found that Chinese netizens who were female, lived in rural areas, had less health literacy, searched less frequently for online information, and had high trust in web-based information released by celebrities were more likely to be misled by misinformation on social media, since they were more likely to easily change their health behaviors without fact-checking and cross-verifying web-based information. These findings have practical implications for the government, health organizations, and health practitioners in designing and implementing health promotions and interventions in similar pandemics. Netizens with the aforementioned characteristics should be informed about the risk of misinformation and the strategies for verifying the accuracy of web-based COVID-19 information to protect them from using counterfeit, inappropriate, or unsafe preventive measures. More technical and policy efforts are needed to further address the dissemination of misinformation on social media.

## Appendix

Multimedia Appendix 1 Survey questionnaire.

## Abbreviations

AI: artificial intelligence
aOR: adjusted odds ratio
CVI: content validity index
WCH: West China Hospital
