# Supplementary material for: Cross-Verification of COVID-19 Information Obtained From Unofficial Social Media Accounts and Associated Changes in Health Behaviors: Web-Based Questionnaire Study Among Chinese Netizens
Source: JMIR Public Health Surveill. 2022 May 31;8(5):e33577. doi: 10.2196/33577 (PMC9198829; doi:10.2196/33577)
Supplement: Multimedia Appendix 1 [file publichealth_v8i5e33577_app1.docx]

**Multimedia Appendix 1. Survey questionnaire.**

(Translated from the original version in Chinese)

| Please answer the following questions in the spaces below. To select an answer, draw a "√" in the corresponding box. | | | | | | | |
| --- | --- | --- | --- | --- | --- | --- | --- |
| ***Sociodemographic characteristics*** | | | | | | | |
| Gender | □ Male □ Female | | | | | | |
| Age (in years) | □ <20 years old | | □ 20-29 years old | | | □ 30-39 years old | |
|  | □ 40-49 years old | | □ 50-59 years old | | | □ 60-69 years old | |
|  | □ ≥70 years old | |  | | | | |
| Education | □ Junior high school or below  □ High school/technical secondary school/vocational high school  □ Junior college  □ Undergraduate degree  □ Master’s degree or above | | | | | | |
| Occupation | □ Student  □ Staff member in government  □ Healthcare providers  □ Staff member in a company  □ Self-employed entrepreneurs  □ Other | | | | | | |
| Current residence | □ First-tier cities (Beijing, Shanghai, Guangzhou, Shenzhen, etc.)  □ Second-tier cities (Chongqing, Tianjin, provincial capitals, etc.)  □ Other cities  □ Rural areas | | | | | | |
| **Perceived health status** | □ Good  □ Medium  □ Poor | | | | | | |
| **Perceived health literacy** | □ High  □ Medium  □ Low | | | | | | |
| ***Use of social media*** | | | | | | | |
| **Which of these social media outlets are you using to search for COVID-19 information?**  [Multiple choice question] | □Government agency channels, such as the Chinese State Council  □Professional news media channels, such as Sina Release  □Healthcare media channels, such as the Centers for Disease Control and Prevention  □Medical institution channels, such as West China Hospital  □Celebrity channels, such as famous movie stars | | | | | | |
| **Time spent on social media per day (hours)** | □ time ≤1  □ 1<time ≤3  □ 3<time ≤5  □ 5<time ≤7  □ time>7 | | | | | | |
| **Frequency of browsing information related to COVID-19** | □ Rarely  □ Sometimes  □ Often | | | | | | |
| To what extent do you trust the sources of information related to COVID-19 spread on social media?  [1-5, from low to high] |  | 1 | | 2 | 3 | 4 | 5 |
|  | Government agencies |  | |  |  |  |  |
|  | Professional news media |  | |  |  |  |  |
|  | Healthcare media |  | |  |  |  |  |
|  | Medical institution |  | |  |  |  |  |
|  | Celebrities |  | |  |  |  |  |
| ***Knowledge about COVID-19*** | | | | | | | |
| Which transmission routes are feasible for the novel coronavirus?  [Multiple choice question] | □ Droplets (correct answer)  □ Airborne (correct answer)  □ Contact (correct answer) | | | | | | |
| Which of the following is not suitable for preventing COVID-19 in the choice of masks? | - Gauze mask (correct answer) - Disposable medical mask - Medical-surgical mask - N95 protective mask | | | | | | |
| Which of the following statements is incorrect about the use of masks? | - If conditions permit, populations in dense areas should change their disposable masks around four hours - Once contaminated, the mask should be replaced as soon as possible - Avoid touching the inner face of the mask with your hands - Cotton masks resist the coronavirus better than medical masks (correct answer) | | | | | | |
| Which of the following measures is effective in preventing COVID-19? | - Rinse with light saltwater - Sauna, steaming - Drinking alcohol - Wearing masks (correct answer) | | | | | | |
| ***Did you change health behaviors based on information on social media?*** | | | | | | | |
| Will you change your behavior according to the COVID-19 information from social media? | - Yes - No | | | | | | |
| ***Did you cross-verify COVID-19 information on social media?*** | | | | | | | |
| Will you verify the source and authenticity of COVID-19 information on social media? | - Yes - No | | | | | | |
